# The Lifting Cure

**Published:** 1868-11

**Authors:** 


### THE LIFTING CURE

Is the latest discovery by which everybody and "the rest of mankind" are to be cured of all manner of diseases, not only before, but after, *medicines have failed.*

That exercise is indispensable to good health; that the benefit to be derived from it is proportioned to the good judgment used in employing it, are old and oft repeated health maxims: that it will ever be employed to cure scarlet fever or the measles, we doubt.

We have seen it applied in a gymnasium in New York, years ago—in which there was for a time, a scientific instructor—in such a manner as to produce remarkable results in restoring persons to health who had become debilitated and otherwise run down, as they say; and we are fully prepared to acknowledge, that under proper instruction, there is great benefit to be derived, by most people, by the use of the gymnasium; but the misfortune is, that now-a-days, there are but very few such teachers to be found, and the gymnasium, in most cases, has degenerated into a training school for those who are ambitious to jump further than their neighbors, or to turn more summersets or who wish to become circus-actors.

Our theory of *cure* is, never to *need* it. So live, that you will never be sick. It is far easier to do this than it is to cure you after you have lost your health.

"I don't believe it," people cry out; "every one must be sick sometimes."

Reader mine, study the laws of life and health and *obey* them and you will soon be able to snap your fingers at the doctors, the lifting cure, and the Turkish Bath cures.

Did you ever think of the number of people who are making their living out of those who are either sick or who imagine that they are? The doctors, regular and irregular, the quacks and druggists, wholesale and retail? and of the immense quantity of trash that is daily gulped down by gullible people? The amount of money which is paid for medicines in any one day in the city of New York would pay the expense of all of our public schools for a whole year! and for all the good that comes from it, nine-tenths of it might as well be thrown into the river!

All *cures* that depend upon exercise have this recommendation—and that no little one—that they dispense with drugs.

"Throw physic to the dogs!" was a wise injunction.

